# "The University Lives Anxiety and De-pression": Diagnostic Uses and Affective Negotiations in Mental Health Care Services for University Students in Chile

**DOI:** 10.1007/s11013-025-09905-8

**Published:** 2025-04-01

**Authors:** Angela Cifuentes, Esteban Radiszcz, Francisco Ortega

**Affiliations:** 1 Departament of Humanistic Studies, Universidad Técnica Federico Santa María, Santiago, Chile; 2 Medical Anthropology Research Center, Universitat Rovira i Virgili, Tarragona, Spain; 3 Transdisciplinary Laboratory on Social Practices and Subjectivity, Faculty of Social Sciences, Universidad de Chile, Santiago, Chile; 4 Catalan Institution for Research and Advanced Studies (ICREA), Barcelona, Spain; 5 Medical Anthropology Research Center, Universitat Rovira i Virgili, Av. Catalunya 35, 43002 Tarragona, Spain

**Keywords:** University mental health, Diagnostic labels, Affective negotiations, Anxiety, Chile

## Abstract

The expansion of mental health discourses within the university has attained global relevance over the course of the past decade. This article focuses on the Chilean case, exploring the diagnostic uses and affective negotiations on campus. The findings presented are part of a broader qualitative research that examined the interrelations between the neoliberal restructuring of the Chilean university, the modes of anxious affection among students, and the strategies implemented by university mental health services. We argue that, although the neoliberalization of higher education in Chile has driven normative and subjective transformations, the phenomenon of university mental health involves students’ agency. Our findings demonstrate that, for both mental health professionals and students, university life serves as a "catalyst of anxiety." Despite the existence of individualized diagnostic conceptions, they also allude to the inequalities inherent in the Chilean educational and health systems. We state that diagnostic uses involve strategies that students and professionals deploy to respond to the demands of adjustment/integration to universities, and even facilitate the possibility of re-imagining futures in the face of experiences of failure. Diagnostic uses engage affective negotiations in everyday situations, thereby configuring university life as a dynamic environment, subject to potential and permanent transformations.

## Introduction

Between April and June 2019, a group of students from Chilean universities engaged in a protest demanding the implementation of measures to address the issue of mental health problems among students. One of the banners raised read, “The University Lives with Anxiety and *De-presión*.”^1^ This allusion to the academic overload, the lack of responsiveness from the authorities, and the scarcity of specialized mental health services for students reflected the underlying issues that the protestors sought to highlight. The hyphenated word *de-presión* (de-pressure) implied a multitude of pressures and moral conceptions of *university life* that, when intermingled with mental health labels, resulted in the formation of specific ailments. Life on campus was evoked to illustrate instances where emotional experiences are imbued with heightened intensity and encoded within the language of mental health. These facts resulted in a collaborative effort between students, faculty, and professionals from the student wellbeing services. The use of diagnostic categories not only facilitated the recognition of student suffering but also led to the implementation of concrete measures, such as periods of academic recess, which were referred to by students as “anti-suicide weeks.” At that time, a considerable number of surveys and epidemiological studies were conducted, which revealed a significant prevalence of suicidal ideation and anxious-depressive symptomatology among Chilean students (López et al., 2024).

Although the phenomenon is not new (Crook, 2020), over the past decade mental health in the university setting has gained global relevance. This has led to an increase in attention and discussion of the subject under a rhetoric of crisis (Callard et al., 2022; Shackle, 2019). This concern has been corroborated by research indicating high prevalences of anxiety and depression among the student population (January et al., 2018), as well as a progressive worsening of these conditions (Auerbach et al., 2018; Lewis & Bolton, 2023). In the context of the global pandemic of COVID-19, studies have indicated an additional deterioration in the mental health of university students (Xiong et al., 2020), a situation that has also been observed in Chile (Azún et al., 2022; Mac-Ginty et al., 2021).

Recent research has indicated that the rise in mental health issues among college students may be attributed to a number of factors, including the introduction of measures to reduce the stigma surrounding mental illness on campus, increased screening for pre-existing psychological vulnerabilities, academic demands, and so-called "social risk factors," such as racial, class, gender, and ability disparities, and financial difficulties associated with pursuing higher education (Alsopp et al., 2023). Nevertheless, it is challenging to ascertain whether the observed increase in mental health issues among college students is a direct consequence of the global proliferation of mental health labels in the context of new political, technological, neurobiological, ecological, and other forces that have the potential to overwhelm the interpretative frameworks that have hitherto been in place (Béhague & MacLeish, 2020; Lovell, 2020). Therefore, the cultural and political work underlying the objectification of mental health discourses on a planetary scale and the new emerging epistemes are interwoven with local cultural projects with diverse and specific applications (Béliard et al., 2022; Ortega, 2020).

The conceptualisation, utilization, and ramifications of diagnostic labels in the Chilean university context are manifold and multifaceted, as evidenced by recent research conducted in the USA (Alsopp et al., 2023) and the UK (Armstrong et al., 2023). In contrast with the oppressive language typically attributed to labels and mental health discourses in general, and despite the fact that influential publications have examined diagnostic labels as a source of stigma, ethnographic studies interested in the phenomenon of university mental health reveal a dynamic scenario in which these labels shape the cultural landscape and acquire social life (Armstrong et al., 2023).

A number of studies concur that poor student mental health is shaped by broader social forces linked to the neoliberalization of universities. These include the imposition of market-driven regulations, competition, and metrics (Cannizzo & Osbaldiston, 2019; Valero et al., 2019). However, these forces take shape in different ways according to the local aspects of university cultures (Gallardo et al., 2019). For example, the Anthropology and Mental Health Interest Group (AMHIG) of the US Society for Medical Anthropology has stated that student distress is closely linked to the management of universities as "academic enterprises" and the political economies of higher education (Fletcher et al., 2022). Similarly, studies conducted in the United Kingdom have identified a correlation between the promise of social mobility, social inequality, and the expansion of university enrollment in recent decades with the emergence of mental distress (Cant, 2018).

The results of the World Mental Health Surveys International College Student (WMH-ICS) indicate that the transformation of the educational system may pose a significant risk to the mental wellbeing of these young people (Evans-Lacko & Thornicroft, 2019). Social science studies in mental health have highlighted the necessity to move beyond mere numerical data and to critically examine the efficacy of prevailing approaches that rely on individualized and reductionist interventions. These include the teaching of resilience skills, coping strategies, the implementation of digital devices, mindfulness, and others. However, there is a growing recognition that these approaches are often disconnected from the evidence concerning the role of complex environmental, institutional, social, political, and cultural factors (Alsopp et al., 2023; Callard et al., 2022). Therefore, situating the issue within the university context necessitates consideration of the fact that the use of mental health labels on campus is not always a source of stigma and can convey different meanings, enabling institutional recognition of issues that fall outside the scope of categories such as "exhaustion," "overwhelmed," or "stress," which do not enter the university bureaucratic system (Armstrong et al., 2023). In other words, mental health labels would have a distinct moral economy, differing from other forms of naming experiences (Fassin & Rechtman, 2007). This would entail the development of social life and the involvement of diverse practices.

A review of the literature on the socio-anthropological studies of mental health (Ehrenberg & Lovell, 2001; Fassin & Rechtman, 2007; Martin, 2009) indicates that the challenge of investigating the use of diagnostic labels is less about identifying causalities than it is about characterizing the relationships between discourses, modes of expression, and practices, including their social, cultural, historical, and political dimensions (Cottet et al., 2023; Hacking, 1999; Huertas, 2011). From this situated perspective, we propose that the relevance of university suffering translated into diagnostic categories provides insights into how mental health globalization processes and local scenarios mutually shape each other. This involves not only discursive and epistemic aspects, but also *the ways in which they affect people* and institutions and *how* they respond to these effects.

This article examines the ways in which both university mental health care professionals and students engage with the most common mental health labels on campus, and how these lead to the emergence of *specific modes of affectation and negotiation*. In other words, our analysis will focus on the ways in which these labels are used, conceived of, and managed, and how they are imbricated with the demands of university life. This article will display the relationship between diagnostic uses on the Chilean campus and the normative configurations and types of demands characteristic of university cultures. Despite the overwhelming majority of our interviewees indicating an intensification of anxiety during the university period, both students and professionals concur that anxiety is a label that encodes a pervasive affect, encompassing expectations of success or social mobility, projects, ideals, and the social relations that institutions promote, in addition to the academic strains.

The following sections will first situate the Chilean context and examine the evolution of "university mental health" in recent decades. Second, we will present the methodology used in the study. Third, we will discuss our findings, starting by examining how our interlocutors conceptualize the category of *university life* as a "catalyst of anxiety" in the context of performance and social integration pressures. Next, we examine the pragmatic purposes served by the uses of diagnostic labels. Our findings evince that, in general, for both professionals and students, the use of diagnoses does not necessarily entail epistemological, ontological, and/or etiological commitments. Then, we will delineate the manner in which diagnostic labels are integrated into practices of accompaniment and re-imagination of alternatives in the context of experiences of failure. Finally, we will illustrate, through a practice we have named *contesting experiment*, how diagnostic labels facilitate *affective negotiations* involving the acknowledgment and problematization of "the social'' as a pivotal aspect of the university experience.

## The Evolution of University Mental Health in Chile

The neoliberalization of Chilean universities was promoted during the dictatorship with the General Law on Universities (1981), which facilitated the privatization and establishment of "educational services" focused on the production of *human capital*, aligning with the ideological vision of the military regime. At that time, there were eight universities in existence, comprising two public and six private institutions with state subsidies. The number of universities has now reached sixty, with nearly eighty percent of these being privately owned. Indeed, over the last few decades, a significant number of students from disadvantaged socioeconomic backgrounds have gained access to higher education, creating a new horizon of possibilities based on the promise of social advancement. This promise was reinforced during the return to democracy through the implementation of a system of student loans (Crédito con Aval del Estado), which resulted in a significant number of young people becoming indebted. In this context, student demonstrations occurred in 2006 and 2011, during which protesters demanded an end to profit and the implementation of a free, public, and quality education system. These demonstrations contributed to the creation of the free education policy initiated by the Michelle Bachelet government in 2014. The aforementioned policy, implemented in 2016, did not ultimately encompass the concept of universal free education. Instead, it focused on the most economically vulnerable 60% of the population, as determined by income distribution (Rodriguez et al., 2023). Subsequently, the surge in enrollment of young individuals from disadvantaged backgrounds and the intensification of expectations of equality have given rise to impassioned debates and new student demonstrations, which have encompassed matters pertaining to access to mental health care at universities (Jáuregui, 2022).

Since their inception, Chilean universities have implemented comprehensive support mechanisms for students, with a particular focus on ensuring their emotional wellbeing. During the 1960s and early 1970s, the so-called "Student Wellbeing Secretariats" were responsible for the promotion of policies designed to enhance student support and encourage participation. Subsequently, in the context of political persecution and state violence during the military regime, the universities were intervened in and the "Student Services Departments" were established. This introduced a wellbeing vision based on a corporate model. Consequently, a new semantics of student wellbeing has emerged, centered on individual responsibility and the logic of "management" (Cifuentes, 2023). Thus, alterations to student wellbeing apparatus were influenced by the diversification of human capital in higher education, whose discourses and strategies transformed the university ethos and academic work (Thayer, 2019).

The student demonstrations in Chile regarding mental health issues that occurred between April and June of 2019 represented a significant shift in the manner in which student demands were addressed, this time utilizing the language of mental health. Subsequently, discussions surrounding university mental health have been characterized by a dichotomous approach, with debates centering on the contrasting themes of individual and community, mental distress and social suffering, and student clientelism and politicized malaise. This has resulted in a neglect of the everyday experiences and social interactions that occur on campus (Cifuentes, 2023). For instance, it has been proposed that the expression of distress under the rubric of mental health is a consequence of the depoliticization of university malaise facilitated by the ascendancy of "psi" knowledge (Apablaza, 2020).

While there is a general consensus that the process of university neoliberalization has resulted in normative and subjective transformations (Fardella, 2020), we argue in light of our material that the phenomenon of mental health on campus, despite being situated in a context of individualization of distress, involves at the same time forms of agency that challenge or strain the hypothesis of “depoliticization” advanced by Apablaza (2020). Such a reading assumes that diagnostic labels explain students’ distress as a result of a managerial and psychologizing logic, in which professionals would be no more than representatives of forms of domination driven by academic institutions. As we will see in the results, our research indicates that diagnostic configurations and practices are more complex and nuanced. While “the neoliberal ethos is manifest in the actions and emotions of university employees” (Valero et al., 2019), we believe that a nuanced understanding of the labor of interpretation requires a critical distance from totalizing readings of neoliberal capitalism (Latour, 2014). This approach allows us to examine the ways in which individuals within universities are prompted or encouraged to act in accordance with or deviate from socially prescribed norms (Berlant, 2011; Stengers & Pignarre, 2007).

## Methodology

This article presents findings from a broader qualitative research that examined the interrelations between the neoliberal restructuring of the Chilean university, the modes of anxious affection among students, and the strategies implemented by university mental health services. It is noteworthy that the research was influenced by a series of social events in Chilean society. These included demonstrations around university mental health (April to June 2019), the social unrest of October 2019, and the global pandemic of Covid-19 between 2020 and 2022. These events influenced the initial design of the study, and the methodology evolved in response to the challenges presented by the social context.

This study included a set of criteria for the selection of institutions, taking into account various factors such as the type of university (based on selectivity, level of demands, student profile, and geographical location), with the aim of encompassing three main configurations that reflect the diversity of the Chilean higher education context. These configurations include a Traditional University with high demands and academic selectivity located in Santiago; a Traditional University with medium demands located in the Central-South Zone of the country; and a Non-Traditional Private University with low demands located in Santiago. Following the requirements of the ethics committee, we had to anonymize all universities and interlocutors involved. To this end, we decided that, rather than distinguishing between public and private institutions, a more appropriate classification would be that of 'traditional' and 'non-traditional' universities. This classification is based on the membership of the institutions in question to the Council of Rectors of Chilean Universities (CRUCH). This organization defines a traditional university as an institution with the greatest prestige and tradition of excellence, encompassing all public universities in the country and a selection of private ones. Non-traditional universities are defined as private institutions with low or moderate levels of demand and selectivity, and a student profile comprising primarily middle-class students and those from disadvantaged socioeconomic sectors.

In April 2021, the ethical aspects of the research were formally approved and our fieldwork began. This phase entailed the conduction of semi-structured interviews (Hammer and Wildavsky, 1990) with mental health service professionals and ethnographically inspired interviews (Guber, 2001; Spradley, 2016) with university students. The first set of interviews was designed to investigate the discourses of professionals concerning their practices, negotiations, and interpretations of mental health problems in university students. The most commonly used diagnostic labels were also examined, as well as the professionals' experiences in treating students, their achievements, difficulties, and challenges. Conversely, the interviews with students were conducted without a predetermined guideline, instead, questions were posed to elicit narratives around their experiences and emotions. The interviews explored the interactions with professionals regarding diagnoses, encompassing the scope of social relations, values, and demands that permeate university life.

Due to the prolonged suspension of in-person classes and stringent covid-19 prevention protocols in Chile, the interviews with professionals and students were conducted online. As the professional interviews proceeded, an internal call was distributed at each institution, inviting students who had experienced "anxiety and/or distress problems" during their tenure at the university to participate. The interviewing process was concluded in August 2022. A total of 21 professionals and 44 students were interviewed. Most of the professionals were clinical or educational psychologists, social workers, and psychopedagogists, all of whom held management and direct care positions in university mental health care services. The students' ages ranged from 18 to 25 years old. With respect to the sociodemographic characteristics of the students, the study encompassed a range of variables, including gender, degree type, and the status of being the first generation in their family^2^ to pursue a degree (see Table 1). These conditions were held constant across universities, except at the Traditional University of Santiago, where only female students expressed interest in participating, despite the dissemination of the call in various faculties, including those traditionally masculinized.^3^ The preponderance of first-generation students in the study aligns with the prevailing demographic patterns in Chile, where official statistics indicate that 70% of the student population falls within this category (Flanagan-Bórquez et al., 2023; Higher Education Information System [SIES], 2012). It is worth noting the heterogeneity of the university degrees of the students participating in our research: medicine, occupational therapy, nursing, dentistry, sociology, social work, psychology, pedagogy, engineering, performing arts, among others. Since this article deals with the uses of mental health diagnoses, we decided to exclude psychology students, to avoid bias regarding the specific mental health knowledge of these students.

**Table 1:** Sociodemographic description of the students interviewed

	Traditional university (Santiago)	Traditional university (Central-South Zone)	Non-traditional private university	Total
Women	14	8	9	31
Men	0	7	6	13
First generation in their family to pursue a degree	9	13	9	33
Not first generation	5	2	5	12
LGBTIQ+	1	0	1	2
Ethnicity	1	0	0	1
Migrant	0	1	0	1
2nd university degree	1	2	6	9
Diagnosis before university	1	3	2	6
Diagnosis at university	5	2	0	6
Not diagnosed	8	10	14	33
Total	**14**	**15**	**15**	**44**

Our fieldwork revealed that only a minority of students who had been formally diagnosed received their diagnosis prior to entering university (see Table 1). Most students had not received a formal diagnosis, even if they reported experiences of anxious and/or depressive episodes of varying intensity during their university period. All participants demonstrated a high level of familiarity with the language of mental health and had conducted online research into their symptoms. This prompted us to examine the particulars and utility of diagnostic uses in the context of university life, encompassing the *ways in which individuals are affected*, the social dynamics at play, and the affective negotiations involved.

To analyze all the interviews, we first conducted a content analysis (Bryman, 2004). This inductive method of analysis generated descriptive and conceptual categories through which we made a general mapping of the main findings. Based on the specific findings, which in this article refer to diagnostic use, we then conducted a discourse analysis aimed at identifying interpretive repertoires (Potter & Wetherell, 1987; Wetherell & Potter, 1996). This method sought to go beyond regularities or consensus to identify some tensions and variations in repertoires, considering that people's experiences and affects involve situations in permanent and potential change (Potter & Wetherell, 1987), thus involving an analytical approach committed to thinking in the midst of a world in process (Duggan, 2020). The identification of interpretive repertoires in our empirical material consisted of identifying the most frequent uses of language by professionals and university students, that is, their explanations and propositions about diagnostic labels, considering not only the textual nature of the meanings, but also what the discourse of mental health does on campus, including negotiations and agencies that emerge in response to the demands of Chilean university life. Finally, we carry out a synthesis of the results and triangulation of the data with the theoretical discussion.

## Results

### University Life as a 'catalyst for anxiety'

Most professionals from the participating institutions concur that students' problems are "multi-causal," invoking the concept of "social determinants of health." However, many of them emphasize that although some students experience mental health problems before entering higher education, it is the performance demands of university life that act as a catalyst for such problems. The director of the student wellbeing service at the Traditional University of Santiago observed that 90% of student consultations on spontaneous demand are directly linked to the concepts of "competitiveness" and "success ideals," which the university actively promotes. For his part, a psychologist at the Traditional University in the South-Central Zone of the country combines university life demands with specific social and individual "vulnerabilities":

University life can act as a catalyst for the emergence of other vulnerabilities or difficulties. While the academic workload is a significant factor, it is not the sole cause. Many students begin to encounter difficulties, such as struggling to cope with certain courses, which can lead to a decline in self-esteem. As they are unable to meet the demands placed upon them, they may begin to devalue themselves, question their abilities, and engage in self-criticism. This can subsequently affect other areas of their lives, extending beyond the academic domain. Then they say, "I'm not intelligent, I'm going to do badly in life, I won't be able to succeed later on life".

The director of a university psychological support unit at a private university in Santiago indicates that students utilize the support unit "especially during evaluation periods," particularly at the beginning of the academic year and the end of the semester. A psychopedagogist at the university links "academic self-esteem" with "anxiety and concentration difficulties," noting that a significant portion of her work “involves challenging the conventional norms of academic behavior, which are often shaped by the expectations of instructors.” For the professional, the norms involved in performance requirements constitute "rules that one must learn to adhere to," many of which are unfamiliar, particularly to students from disadvantaged backgrounds and public schools. This is a point that is made in all of the participating universities. Individualized conceptions of student anxieties coexist with a recognition that academic life involves normative aspects of university cultures expressed in social relations, and that the university ethos promoted by the faculty is relevant.

The pervasive understanding of student mental health issues through the lens of social determinants of health coexists with an emphasis on individual predisposition. This reflects the view among professionals that healthcare is only justified in the presence of psychological conditions or problems associated to pre-existing vulnerabilities (Alsopp et al., 2023; Armstrong et al., 2023). However, while the concept of mental health and wellbeing for students and professionals often encompasses individual factors such as academic self-esteem, self-care, and personal responsibility, professionals at the three universities recognize student distress in the context of the multifaceted dimensions that shape university life. Indeed, while their conceptions allude to family, biological, and individual factors, as well as a course of illness beginning in adolescence, most of them emphasize the inequalities of the Chilean educational system, the ineffectiveness and inadequacy of public mental health services, and the relevance of considering socioeconomic status, gender, and place of origin (rural or remote area) in understanding students’ experiences and in the management of their psychological distress. In the case of Chile, we posit that this issue is related to two historical elements. 1. Mental health professionals have tended to redefine their approaches, focusing on social stratification and economic inequalities. These historical roots can be traced back to the development of community psychiatry in the 1960s and 1970s and the promotion of gender and multicultural policies by the state in the 1990s (Abarca-Brown, 2024). 2. The historical role of the Chilean feminist movement in the struggle for a dignified life over the last forty years, and the effect of the May 2018 student movement around gender-based violence at universities (Follegati & Ferretti, 2022).

As an illustration, some professionals have expressed reservations about reducing students’ challenges and institutional responses to "individual healthy behaviors," "resilience," or digital technologies for emotional self-management. A psychologist at Santiago’s Traditional University remarks that student suffering is related to structural inequalities reflected in the dissimilar educational trajectories of students at that university. This phenomenon has become more pronounced with the increase in students from disadvantaged socioeconomic backgrounds as a result of policies mandating free access to higher education. He further states that student mental health issues “emerge as a result of underlying issues pertaining to coexistence,” including instances of “gender-based violence” and the “authoritarian” approach adopted by some faculty members and academic authorities toward their students, in addition to the “novel conditions of academic life introduced by the pandemic.”

Both professionals and students indicated that the pandemic had exacerbated mental health issues not only through the direct effects of social isolation, illness, and loss but also, and more profoundly, by deepening the social inequalities experienced by students. Additionally, some students reported feeling a sense of relief and a reduction in anxiety due to the physical distance from the heightened demands of competition and performance in face-to-face university life (Alsopp et al., 2023). Indeed, in all universities, respondents indicated that the pandemic compelled institutions to advocate for enhanced academic flexibility.

Although most professionals and students indicate that university instills new anxieties that are distinct from their previous life experiences, some interviewees elaborate on this notion. The material and spatial aspects of university life are perceived by some respondents as a conduit for the formation of collaborative and supportive networks, which are often absent or inadequate in the context of familial and mental health care structures. It is notable that while students from disadvantaged backgrounds, particularly those who are the first in their families to attend university, experience anxiety about social and academic failure within the educational space, this same space also provides opportunities for them to interact with others, including peers, teachers, and professionals, who not only encourage their resilience in the face of academic pressures but also inspire them to pursue personal projects.

A medical student at the Traditional University of the South-Central Zone of the country notes that, despite the anxiety associated with the pursuit of academic excellence, which is a prerequisite for the maintenance of tuition-free status, interactions with “students from humble or middle-class backgrounds contribute to a less competitive atmosphere than that observed in other universities.” Indeed, the university's geographical location results in a student profile comprising young people from rural areas, the majority of whom are the first generation of university students in their families. In fact, the student reports that receiving friendly treatment and recognition of her intellectual abilities from faculty and classmates has led her to perceive herself as "an intelligent and capable woman," in contrast to her previous academic experiences.

In contrast, a medical student at the Traditional University of Santiago, also the first of her family to enter university, describes an academic culture that is characterized by an “obsession with success” (*exitismo*), competition, and trajectories of academic excellence, which are mostly observed among students from elite schools. For her, entering the university meant a transition from the elation of achieving “what she wanted most in life” to confronting a "very sad, classist environment... a lonely world." This is illustrative of the experience of many students from disadvantaged backgrounds who enter high-performing careers at elite universities. Their experiences of suffering as a result of classroom performance are related to the reproduction of inequalities (Villalobos et al., 2022) in social interactions, particularly in interpersonal contact (Araujo, 2019). It is noteworthy that a considerable number of students pursuing degrees in health-related fields have reported experiencing distress in situations of mistreatment. This finding is particularly troubling given that, despite official discourses of gender equality, inclusion, and wellbeing, the university environment is not as inclusive as it should be and does not meet expectations regarding the acceptance of mental health problems (Kirsh et al., 2016).

### Diagnostic Practices on the Chilean Campus

Most professionals surveyed indicated that their diagnostic practices are primarily driven by the institutional mandate to "respond effectively to the over-demand" for care. In addition, students frequently utilize mental health categories to articulate their experiences of distress within the university setting, irrespective of whether they have received a formal diagnosis. In this context, the use of diagnostic labels on campus does not necessarily indicate a preference for "psi," "neuro," or "biomedical" knowledge. Rather, it represents a strategic practice, a means of navigating the demands of university life and adhering to technical-administrative requirements.

Diagnostic labels serve as *campus technologies*, acquiring diverse meanings and utilities (Armstrong et al., 2023). Our analysis revealed specific diagnostic uses associated with the most frequent labels, namely anxiety and depression. This finding corroborates epidemiological studies addressing university mental health on a global scale, which have identified depressive disorders (18.5%) and generalized anxiety (16.7%) as prevalent conditions (Auerbach et al., 2018). It also aligns with local epidemiological studies (Martínez et al., 2021). It seems plausible to suggest that this may be related to the fact that most professionals are aware of the current state of university mental health studies and are influenced by institutional concerns and expert discourse around the university mental health crisis (Crook, 2020).

However, in contrast to other psychopathological diagnoses that are less frequently mentioned by students and professionals, such as bipolar disorder, personality disorders, eating disorders, drug dependence, and ADHD, the labels of anxiety and depression appear to be associated with specific situations of university life. This is particularly evident when noting that "academic stress" or "cohabitation problems" do not always guarantee institutional attention. Moreover, the expansion of institutional resources dedicated to the establishment and specialization of university mental health care services has coincided with the quantification of institutional quality based on student performance and retention metrics. In this context, university mental health services are positioned as facilitators of integration. Indeed, the accreditation requirements in the participating universities have exerted pressure on the strengthening of mental health care services, and the use of labels is consistent with this context. As a psychologist from the Traditional University of the South-Central Zone notes,

I think there has been a lot of advancement in the integration of mental health initiatives within universities, not only here. It has been something generalized that universities have begun to give importance to mental health and to see that this also has a direct impact on the performance and permanence of students at university. We should not be naïve about the fact that there may also be a motivation behind the way in which universities have been focusing on mental health.

Labels involve strategies of power deployed in the relationship with institutions and social environments that transforms a student with a diagnosed condition into a recipient of mental health care, rendering them legible to the institution (Crook, 2020). This transformation also subjects them to intervention aimed at facilitating their adjustment to academic requirements. Indeed, those who appear to be “mentally well” would be at a disadvantage compared to those who are diagnosed with a mental illness (Armstrong et al., 2023). Furthermore, in the Chilean context, a mental diagnosis becomes a focal point for institutions, as it is perceived as a *risk factor for student attrition*, thereby increasing the value and demand for university mental health services. As stated by the professionals interviewed, the so-called student retention rate is one of the institutional quality evaluation criteria, providing insight into the efficiency of the university's wellbeing, inclusion, and integration strategies (Cifuentes, 2023). In other words, the management of mental health on university campuses in Chile represents yet another indicator for the evaluation of quality and excellence in higher education. The logic of performance and results quantification imposes political-economic norms on the university space (Fardella, 2020).

Conversely, a significant proportion of students who utilize university mental health services are users of the public mental health system and hail from educational institutions situated within socioeconomically disadvantaged sectors. The quality of education in these institutions is markedly inferior to that of private schools, where students are more likely to pursue traditional university pathways that offer greater prestige and resources. Indeed, the high demand for psychological care at universities appears to be an expression of a political economy of student suffering.^4^ As a first-generation student from the Private University points out,

In Chile mental health for us poor people do not exist. We have to wait a long time to be called. That's very bad because you go, and they tell you: 'I have an appointment for another month', so you say: 'in another month I'll do better (*tiro para arriba*) or I'll do things or I'll look for something that can help me'. The university has psychological support services, but there are a lot of people seeking mental health help, so they are overwhelmed.

In Chile, the expression *tirar para arriba* (literally, "throw up") has two distinct meanings: (1) To encourage oneself; (2) To enhance one's socioeconomic standing or to embark upon a venture. At this juncture in the interview, the student refers to the first interpretation, although she subsequently alludes to the second, particularly her aspiration to "be someone in life." This expression, which is frequently employed, represents university studies as a pivotal factor in attaining social mobility. In this context, a diagnosis is a prerequisite for accessing support services and for differentiating between students who can afford psychological treatment outside the university and those who cannot. In other words, without a specific psychopathological diagnosis, students are unable to justify their demand for support on campus (Alsopp et al., 2023; Kirsh et al., 2016), and professionals are unable to argue the necessity of care for these students. This illustrates that the pervasive notion in academic institutions that psychological distress is primarily the result of individual predisposition is closely linked to the conviction that healthcare is warranted when biological or familial predisposing factors are present, particularly when resources are limited. Therefore, the deployment of individual and biomedical models seems to be more strategic than conceptual (Alsopp et al., 2023; Armstrong et al., 2023; Deacon, 2013).

The majority of the psychologists we consulted indicated that their diagnostic procedures serve to delineate the extent of student distress, which is not, however, determined by psychopathological criteria, but rather by the student's support and/or care networks. Indeed, diagnostic practices and therapeutic decisions are based on the availability of care networks, with the socioeconomic level of each student being a determining factor. In the context of the pandemic of COVID-19, many professionals indicated that they were compelled to see nearly all of the students who sought their assistance, with limited avenues for referral to public healthcare services due to prolonged waiting lists. Consequently, the strategic use of mental health labels involves pragmatic negotiations (Ortega, 2020) situated in the contingency of suffering, worries, and burdens that must be conveyed through the rhetoric of mental health to be eligible for entry into the care network.

### Re-imagining Alternatives to Failure

Our findings indicate that mental health labels not only shape identities or act as a way of coping with "hermeneutic injustice" (Fricker, 2007, in Armstrong et al., 2023), but above all, they promote pragmatic operations. Indeed, in some cases, diagnoses are incorporated as part of students’ identity. However, our material evinces that for most students, diagnoses of “anxiety” or “depression” involve pragmatic rather than identity operations, being assumed as situational conditions and specific to the demands of university life. These diagnoses enable the re-imagination of alternatives to experiences of failure, especially in rigid and highly demanding academic contexts. In other words, they do not only involve technologies of "self-creation and self-discovery" (Armstrong et al., 2023), but also play a role in negotiations between students and professionals in situations of significant distress. Therefore, they encourage actions that seek relief despite contravening the institutional demands of "retention." For instance, a diagnosis is employed in the preparation of psychological reports as a means of substantiating, justifying, and facilitating a temporary suspension, change of career or university, and providing rationale for mental health-related considerations. As articulated by a student at the Traditional University of Santiago:

I remember the psychologist explaining to me that he needed to write a diagnosis so that I could drop out of university and he explained that 'depressive anxiety disorder' was just a way of categorizing what I was feeling, but it didn't mean that I would be like that for the rest of my life or that it would determine a change in me. I remember him saying 'think of it more as a bureaucracy, rather than something you're going to have to carry forever'. So, it wasn't just 'I diagnose you and let's move on'; he took it on board without me telling him that it might be hard for me to accept.

Diagnostic negotiations such as this one evince that labeling practices on campus are not always conducive to addressing the intense experiences of distress and life decisions that professionals face when attempting to imagine alternatives to academic failure with their students. This can result in not only a reconfiguration of life projects but also their understanding and use of diagnoses. In the case of the aforementioned student, the encounters with the professional enabled a new way of understanding diagnoses. This new understanding posits diagnoses as situational categorizations that do not necessarily determine a fixed ontological quality. This is particularly pertinent when considering the specific nuances of diagnostic uses and experiences of suffering across different academic fields and universities. In a similar vein, as observed by Ortega-Vega (2021, in Armstrong et al., 2023), students in the humanities tend to exhibit a more favorable disposition toward publicly using diagnostic language and are more inclined to express their emotional experiences. Conversely, health degrees’ students tend to conceptualize mental health labels in terms of functional impairment, leading to heightened self-criticism of their abilities and a greater fear of discrimination, particularly given the prevalence of stigmatizing attitudes among healthcare professionals (Horsfall et al., 2010).

Diagnostic negotiations have the potential to alter expectations and reshape one's presence in the world. This is because they facilitate the conceptualization of alternative futures (Anderson et al., 2018; Cornish et al., 2016). In the context of our fieldwork, the experiences of anxiety and depression can be understood to encompass a range of practices, meanings, and modes of affect regulation. As previously stated, mental health labels are not always associated with medical authority and actors within a university space can mobilize them for a variety of purposes. In contrast to the perception of diagnostic labels as oppressive or stigmatizing, some students view them as a potential avenue for less punitive disengagement. The decision to withdraw from or temporarily suspend a highly sought-after career path—as exemplified by the choice to leave a degree program with high social prestige—can facilitate a process of vital persistence when undertaken in collaboration with the institution. As one engineering student attests, "Thanks to the support I was able to realize that failure is not the end of the world."

Practitioners and students’ observations indicate that labels serve less as instruments of governance than as *ways of grievance and redress* (Armstrong et al., 2023; Béhague & MacLeish, 2020). Our interlocutors demonstrated how mental health labels frequently function as coping mechanisms to prevent despair, contributing to the development of interventions that facilitate repair and envision alternative possible worlds (Anderson et al., 2018). This is achieved by fostering questioning and attention to concrete, sensitive situations as a form of experimentation (Stengers, 2019).

### Affective Negotiations and Contesting Experiments

Our interlocutors revealed the category of anxiety with a range of tonalities and intensities, indicating a situational “affect”^5^ pertaining to the specific demands of university space and time. University life was perceived as a dynamic space of encounters, tensions, synergies, and discontents. In this context, diagnostic labels circulate in a field of grievances, expectations, and specific moral conceptions. Most professionals did not portray themselves in their discourses as passive recipients of the demand for attention. Neither did they present themselves as mere agents of social control who diagnose unthinkingly or who apply rigid intervention guidelines aimed at taming student subjectivities. They articulated their concerns, fatigue, and commitments in a context where the cultural aspects of mental health care are often silenced by the organization (Ortega and Wenceslau, 2020). They spoke from their bodies, which are arranged in an assemblage of norms, affects, and demands that affect them in everyday encounters and in the modes of institutional (self-)surveillance of their practices.

Their practices, as moral subjectivations (Fassin, 2006; Hasenfeld, 2000), are deployed in response to the institution's demand to adjust, integrate, and provide wellbeing, thereby neutralizing politicized expressions of student suffering. However, some practitioners observed a concern about stigmatizing students or adopting a paternalistic approach that would result in a loss of agency. Such critical positionings occasionally manifested as descriptions of interventions that we conceptualized as *contesting experiments*, given that they emerged in the context of a world in process (Stengers, 2019), namely, a university life in potential transformation. A professional from the Traditional University of Santiago described a group intervention that, in a random and experimental way, allowed him to transform his understanding of student suffering. This illustrated the possibility of implementing alternative views to the official guidelines, which are centered on individualizing psychological interventions. In response to the recurring need for assistance from students presenting with anxious symptomatology and its correlation with difficulties in coping with academic demands and social integration, he proposed a group workshop attended by students hailing from disadvantaged and peripheral sectors of the city. He states,

They all went to public schools and lived within an hour and a half to two hours away from the university. For them, observing and feeling like foreigners in a place where their classmates all seem to be geniuses made us realize that, in reality, they are not all geniuses, but that their classmates came from other schools and other backgrounds. So, at first, they all thought that the problems were their own, that the merit was individual. But in the end, they realized there were other nuances [...] What´s interesting about this group, which came out purely by chance because it was not intentional, the type of students that arrived [...] They recognized that their contextual circumstances had freed them from something that had been very present but that they couldn't name.

The professional describes how the planned workshop on time management and anxiety management evolved into a space where students could discuss their sense of alienation in relation to the university's ethos, characterized by an emphasis on excellence, performance, productivity, and competition. The experience of inferiority, as evidenced by an inability to respond to academic requirements in a manner comparable to their "genius" peers, manifested anxieties that the professional elucidated in relation to their life trajectories and peripheral positions with respect to the typical student at that university, where a significant proportion of students hail from academic excellence-oriented schools. Furthermore, he underscores the institution's role in fostering specific forms of relationships, particularly between faculty and students, as well as between peer groups. This is done by promoting competition and differentiation based on academic performance and social class.

Such practice, which originated from institutional concerns regarding the diagnosis of anxiety, created an opportunity for *affective negotiation*. This experimental practice challenged the conventional wisdom that anxiety can be managed effectively through the efficient use of time as well as the official practices promoted by positive psychology and emotional management. He states, “what motivated them was to recognize that they began with a disadvantage and sought how they can overcome that distance, because it did not make sense to them to say ‘think positive, it’s not all that bad', these discourses of ‘being in fullness’ (*vibra alto*) did not convince them.” The identification of the contextual conditions resulted in a willingness to "to talk about the differences and recognize that they will be present in the path they chose." This, in turn, led to the pursuit of strategies to secure a position within the university. Additionally, he emphasized the significance of fostering alternative perspectives that challenge the pervasive notion within university culture that academic success is solely determined by individual merit, without consideration of the multifaceted contextual influences, including economic, material, social, cultural, and familial factors.

For the professional, the experience of anxiety was the result of what this group of students perceived as "an impossible task," a (self-) imposed demand encouraged by a university culture that demands "high performance" and to go toe-to-toe with their "genius" peers. In effect, the normative configurations of the university served as a point of departure, rather than an endpoint, in such practice. Rather than facilitating an environment that merely maintains an ethos of excellence, it instead encourages a space of critical reflection on individual merits and a search for collaborative modes of relationship among students.

We propose that diagnostic uses and practices such as the one described above involve *affective negotiations*. With this notion, we underline that when prompted to discuss their experiences, students did not describe anxiety as a fixed diagnosis, but neither as a “natural and unreasonable” emotional experience. Indeed, it is a matter of affective negotiations, since labels—in this case “anxiety”—are the object of a transaction between bodily experiences and the language available and/or circulating within institutions (Ahmed, 2004). In other words, emotions involve pragmatic negotiations that students carry out with themselves and with others, and which are socially and politically inscribed (Despret, 2022). Discussing anxiety justified the necessity to respond to academic demands while mobilizing interpellations to the normalized power relations in university life based on the figure of the "genius" student and meritocracy as a legitimizing principle of individual effort.

Despite procedural imperatives and institutional bureaucracies, practices such as the one described above express a sensitivity to the complexities—normative, ethical-moral, material, time constraints, etc.—entwined in the expectations of success/failure of university students. Thus, some professionals, even in the midst of institutional adversities, deploy alternatives to provide a form of mental health care committed to life (Wenceslau & Ortega, 2022), involving ways of doing and being affected by the question of *how to live* (Lakoff & Collier, 2004). In the words of Béhague and Macleish (2020): “The how and why of psy- hegemony neither begins nor ends with questions of diagnosis, labeling, reductionism, context, stigma, and outcomes … [It] forces an accounting with the multiplicities and contingencies of the psyche’s diverse forms of power" (pp. 8). Consequently, the diagnostic uses on campus act in accordance with normative and moral repertoires, at the same time that they concern affections intertwined with everyday situations, configuring university life as a dynamic environment, subject to potential and permanent transformations.

## Conclusion

Globally and locally, in light of the transformations that have occurred over the past few decades and the processes of neoliberalization that have affected universities, young people view higher education as a promising field of opportunity. In Chile, the extensive array of universities has transformed the pursuit of a degree into a norm or imperative for individuals seeking to "be someone in life" or "succeed," thereby transforming the university space into a context of new demands. In addition to its meritocratic foundations, the university environment is characterized by a normative order that fosters competitiveness, individualism, and an emphasis on performance and productivity. These factors have implications for future employment and life prospects. As we have seen, the university environment is conducive to the formation and codification of emotional responses to diagnostic labels. University life "catalyzes anxiety." In this context, the label "anxiety" often serves as a cover for experiences of loneliness, exclusion, and failure that arise as a consequence of the reproduction of inequalities, particularly in elitist environments such as traditional universities in Santiago and health care and or other high-performance degrees.

The implementation of official management guidelines in university mental health has the effect of isolating the problems as a matter of students’ individuality and inwardness. In effect, official institutional initiatives have served to alleviate student distress by categorizing anxious experiences as a "mental" issue that necessitates specialized expertise and practices. The uses of mental health labels indicates that experiences of distress can be made legible and manageable in a number of ways. The use of idioms of mental health within academic institutions represents a significant and evolving trend. Rather than being confined to the constraints of social stigma or medicalization, these labels suggest that students engage with them in a dynamic and multifaceted manner. Our fieldwork revealed that labels are not fixed entities; they are mobile, not always associated with medical authority, and socially useful. Students adopt diagnostic labels and expand their uses, such as to gain access to student support services or to reimagine alternatives to "failure." This enables the formation of flexible, context-specific strategies of negotiation.

Students deploy multiple uses of diagnostic labels, and most of the professionals interviewed express reservations about the expansion of the institutional concept of mental health as a "niche of a team of mental health professionals." Furthermore, both students and professionals highlight the intricate nature of the issue and the necessity for comprehensive university-wide initiatives. Similarly, some professionals are critical of the demands of "retention" and "adjustment" of students. They question the institutional pressures on the decrease of student desertion as part of the accreditation system, the allocation of resources, and the safeguarding or aspiration of social prestige as a mechanism of profit for universities. It would be erroneous to view diagnostic practices and uses as mere control, surveillance, and standardization devices.

Our fieldwork revealed that the use of diagnostic labels reflects coping strategies employed in response to the pressures of university life. These strategies encompass the specificity of the label, the intensity of the affect expressed or negotiated through a diagnosis, and the normative configurations of each university culture. The label of anxiety, which is sometimes accompanied by the label of depression, is pervasive and intertwined with university pressures and demands. It is a manifestation of the reproduction of inequalities that are expressed in daily interactions on campus, particularly in universities where there is a significant discrepancy in educational trajectories. The group intervention described above illustrates how the daily recognition of structural inequalities in social relations enabled a contestation of the individualized and reductionist conception of university mental health in an institution of high social prestige. This facilitated an appreciation of the recurrent use of labels in conjunction with the imperative to adapt to the university ethos of excellence and competitiveness. It would be beneficial for future research and/or governmental policies to consider the collaborative creation of interventions that address the pragmatic-political dimension of diagnostic labels and the affective negotiations involved in university life.

## Footnotes

1 This is a pun based on the Spanish words *depresión* (depression) and *de-presión* (de-pressure).

2 The concept of the "first-generation student," defined as the first member of their family of origin to pursue higher education, emerged from a series of investigations that illuminated the emergence of a novel university subject. This subject was shaped by historical processes, including the expansion of access to higher education and the intensification of social mobility expectations.

3 This gender bias can be interpreted based on three main hypotheses that are not mutually exclusive: greater expressiveness of discomfort (due to socialization, women have a tendency to verbalize their sufferings more and demand help); greater intensity of university discomfort (women would suffer more because of a highly demanding academic culture, supported by an ethos of historically masculine excellence); finally, women assume greater responsibilities of care (children, family members). The analysis of the gender and intersectional dimension of the empirical material will be conducted in a forthcoming article.

4 The results of the research on which this article is based evince that although students of all social classes use labels and/or are diagnosed with anxiety and depression, in the case of lower-class students those uses acquire special relevance as they are a fundamental condition to be eligible for psychological care within the campus. In this article, we focus on diagnostic uses and forms of negotiation. Giving the limiting word count we opt for a more cross-sectional approach to the material without privileging class-specific analyses. Such a discussion will be included in another article currently in progress.

5 We understand affect as an aspect of both individual and collective experience involving historical-political forces and performative acts that assign value and meaning to bodies, objects, and social spaces, and that configure dynamics specific to a given social context (Ahmed, 2004), in our case, Chilean universities. As mentioned in the methodology, this article is based on the results of a broader research on “anxious economies of university life in Chile,” which addresses the web of affects, social demands, values, and norms.
